# Combined moist airtight storage and feed fermentation of barley by the yeast *Wickerhamomyces anomalus* and a lactic acid bacteria consortium

**DOI:** 10.3389/fpls.2015.00270

**Published:** 2015-04-22

**Authors:** Jenny Borling Welin, Karin Lyberg, Volkmar Passoth, Matilda Olstorpe

**Affiliations:** ^1^Department of Animal Nutrition and Management, Swedish University of Agricultural SciencesUppsala, Sweden; ^2^Department of Microbiology, Uppsala BioCenter, Swedish University of Agricultural SciencesUppsala, Sweden

**Keywords:** moist airtight storage, fermented pig feed, feed hygiene, yeast, lactic acid bacteria

## Abstract

This study combined moist airtight storage of moist grain with pig feed fermentation. Starter cultures with the potential to facilitate both technologies were added to airtight stored moist crimped cereal grain, and the impact on storage microflora and the quality of feed fermentations generated from the grain was investigated. Four treatments were compared: three based on moist barley, either un-inoculated (M), inoculated with *Wickerhamomyces anomalus* (W), or inoculated with *W. anomalus* and LAB starter culture, containing *Pediococcus acidilactici* DSM 16243, *Pediococcus pentosaceus* DSM 12834 and *Lactobacillus plantarum* DSM 12837 (WLAB); and one treatment based on dried barley (D). After 6 weeks of storage, four feed fermentations FM, FW, FWLAB, and FD, were initiated from M, W, WLAB, and D, respectively, by mixing the grain with water to a dry matter content of 30%. Each treatment was fermented in batch initially for 7 days and then kept in a continuous mode by adding new feed daily with 50% back-slop. During the 6 week storage period, the average water activity decreased in M, W and WLAB from 0.96 to 0.85, and cereal pH decreased from approximately 6.0 at harvest to 4.5. Feed fermentation conferred a further pH decrease to 3.8–4.1. In M, W and WLAB, molds and *Enterobacteriaceae* were mostly below detection limit, whereas both organism groups were detected in D. In fermented feed, *Enterobacteriaceae* were below detection limit in almost all conditions. Molds were detected in FD, for most of the fermentation time in FM and at some sampling points in FW and FWLAB. Starter organisms, especially *W. anomalus* and *L. plantarum* comprised a considerable proportion of the yeast and LAB populations, respectively, in both stored grain and fermented feed. However, autochthonous *Pichia kudriavzevii* and *Kazachstania exigua* partially dominated the yeast populations in stored grain and fermented feed, respectively.

## Introduction

Animal feed production is currently one of the largest consumers of energy, non-renewable resources such as mineral phosphorus, and even food grade agricultural products (Pelletier et al., 2011). Furthermore, the hygienic quality of animal feed has a direct impact on hygienic quality of food; for instance, there is a correlation between the number of *Enterobacteriaceae* in animal feed and subsequently in the food chain (Brooks et al., 2001). Fermentations can provide technologies to both decrease the input of fossil resources into feed production and to improve the hygienic quality of feed and food.

Crimping and storing moist cereal grain under airtight conditions requires substantially less energy than the common practice of hot air drying, thus, efficiently reducing both the cost and environmental impact of feed storage (Olstorpe and Passoth, 2011). Moreover, improved nutritional characteristics of moist stored grain have been documented, both in terms of increased concentrations of proteins and essential amino acids, and of decreased contents of the anti-nutrient phytate (Olstorpe et al., 2009). Preservation is based on metabolic activities of naturally occurring microbes, mainly yeasts and lactic acid bacteria (LAB), which remove easily available nutrients, replace oxygen with CO_2_, lower the pH and produce anti-microbial metabolites(Ström et al., 2002; Magnusson et al., 2003; Olstorpe and Passoth, 2011). The yeast *Wickerhamomyces anomalus*, a biocontrol yeast naturally occurring on cereal grain has been shown to efficiently inhibit non-desirable organisms such as molds and *Enterobacteriaceae* in airtight storage systems (Olstorpe et al., 2010b, 2012).

Pig feed fermentation is another example whereby nutritional and hygienic qualities of animal feed may be improved via microbial fermentation. It is generated by mixing a solid phase, usually cereal grain, with a liquid, such as water, whey or wet distillers' grain, and incubating this mixture for several days. During incubation, a population of fermenting microbes develops that decreases the pH to 3.5–4.5; numbers of *Enterobacteriaceae* are also often reduced, and phytate is degraded (Lyberg et al., 2008; Olstorpe et al., 2010a). Fermented feed can improve digestibility of nutrients, feed conversion ratios and daily weight gain (Pedersen and Lindberg, 2003; Lyberg et al., 2006), and reduce the number of coliform bacteria in the gastrointestinal tract (e.g., Scholten et al., 1999; Hong et al., 2009, 2011).

In spite of the obvious advantages of feed fermentations as described above, they also have several drawbacks, primarily due to theire spontaneous nature and thereby unpredictable output. In airtight storage, hygienic problems, predominantly mold growth, can occur if the moisture content of the grain is too low to facilitate a strong fermentation, because preservation then solely depends on the absence of oxygen in the system (Druvefors et al., 2002; Olstorpe et al., 2010c). A water activity (*a*_w_) of approximately 0.75–0.85 is too low to support extensive growth of LAB, but allows the development of storage molds, e.g., *Aspergillus* and *Penicillium* species. This, in turn, creates “hot spots” of increased moisture content and temperature, which may then enable growth of other spoilage organisms (Magan and Aldred, 2007). *Enterobacteriaceae* have usually not been considered a problem in dried cereal grain, as they are supposedly inhibited at low water activities (Asperger and Winterer, 1978). However, we have recently shown that species of *Enterobacteriaceae* can grow on cereal grains with a moisture content of only 14% (Olstorpe et al., 2010b, 2012). Similarly, fermentation of liquid feed is a variable process that does not yield a predictable content of organic acids, thereby generating feed products that may in some cases not be safe or palatable (Beal et al., 2005). This has a negative impact on animal welfare and puts considerable economic strain on the farmer, who cannot trust that the feed is still usable after several days of fermentation.

Recent results showed that it is possible to influence the microbial flora both in airtight storage and feed fermentation by adding yeasts or LAB to the system (Olstorpe et al., 2010a,b; Canibe and Jensen, 2012). It is also very likely that when combining airtight storage and feed fermentation, the storage flora in the grain has an impact on the subsequent feed fermentation. Identifying microbes that can be used both in moist airtight storage and feed fermentation would enable the development of highly efficient universal starter cultures, which, in turn, could streamline handling of these cultures and make their use more attractive to farmers. The yeast, *W. anomalus*, is a promising candidate organism for such an approach. It shows considerable biocontrol activity against both molds and *Enterobacteriaceae* in airtight storage systems from laboratory to farm-scale (Petersson and Schnürer, 1998; Olstorpe et al., 2010b, 2012), and has previously been isolated from fermented pig feed (Olstorpe et al., 2008). LAB such as *Lactobacillus plantarum* or *Pediococcus* spp. are also promising candidates for both fermentation systems, as they are commercially available and belong to the natural microbial flora in both airtight grain storage and pig feed fermentations (Olstorpe et al., 2008, 2010a). Several LAB have also been shown to exhibit anti-mold activity (Magnusson et al., 2003).

This study examines the impact of *W. anomalus* and LAB starter cultures in an airtight storage system on: (a) the microbial populations during storage; and (b) subsequent feed fermentations using the moist airtight stored grain. To obtain results relevant for current technologies and practices, the study was conducted at full scale, on-farm.

## Materials and methods

### Yeast isolate and starter culture

The yeast *W. anomalus* J121 (CBS 100487) used during the study was originally isolated from stored grain. It is stored in the fungal culture collection of the Department of Microbiology, Swedish University of Agricultural Sciences (SLU), Uppsala, Sweden, in glycerol stocks at −70°C. Inoculum was prepared at a yeast-production company, Jästbolaget, Rotebro, Sweden, by growing *W. anomalus* on conventional raw materials (molasses, ammonia, phosphorous, magnesium and vitamins) and drying on a specially designed fluidised bed to exclude carrier material in the product. The LAB starter culture used was the commercial brand Pig-Stabilizer 600 supplied by Vådfodereksperten, Denmark, containing *Pediococcus acidilactici* DSM 16243, *Pediococcus pentosaceus* DSM 12834 and *L. plantarum* DSM 12837.

### Design of the moist storage experiments

Barley (*Hordeum vulgare*) used in the experiment was harvested at the beginning of August 2011 from Nyvla Gård, Uppsala (WGS 84 (lat., long.): N 59° 57.218′, E 17° 31.526′). The moisture content at harvest was 32%. For three of the four treatments, grains were stored overnight, after which they were crimped using a Murska-350 machine (www.kelvincave.com) and packed into eight plastic barrels per treatment, each barrel containing 120 kg grain. These treatments were: M—980 kg of non-inoculated cereal grain containing only the natural microbial flora; W—980 kg of cereal grains inoculated with *W. anomalus* (for the inoculation procedure see Grain Inoculation); and WLAB: 980 kg of cereal grains inoculated with *W. anomalus* and LAB starter culture (Pig-stabilizer 600). In the fourth treatment, D, 940 kg of cereal grain (*a*_w_ 0.96) was dried using hot air to a final moisture content of 13%, after which it was crimped and stored in a container indoors. Treatment M was harvested, crimped and packed first, i.e., before the inoculated grain, to minimize the risk of cross contamination between the treatments. Post-packing, the plastic barrels were closed to exclude air from the storage system. To ensure proper fermentation of the moist grain, the barrels were first opened after 6 weeks. Samples were taken from each treatment at harvest (post-inoculation), after 7 weeks, and then consecutively every 2 weeks during the feed fermentation experiment (see Design of the Feed Fermentation Experiments) until week 19. As this study is part of an extensive feeding trial performed on-farm (manuscript in preparation), samples for microbial enumeration and material for the continuous fermentations could not be taken from all barrels on every occasion; instead all samples were taken from the treatment barrel being emptied at the time.

### Design of the feed fermentation experiments

After the initial 6 weeks of storage, four feed fermentation experiments were started, using the stored grain as raw material. The grain was mixed with water to a dry matter content of 30%. The four treatments were FM (fermented control with grain from treatment M, see above), FW (containing grain from treatment W), FWLAB (containing grain from treatment WLAB) and FD (containing grain from treatment D). In treatment FWLAB, an additional amount of LAB starter culture (2 × 10^5^ cells/g grain) was added at the beginning of fermentation. This was done as advised by the provider, to ensure viability of the cells. The four treatments were fermented initially for 7 days, and thereafter maintained in a continuous fermentation mode, whereby 50% of the material was removed daily for feeding and replaced by an equivalent amount of stored grain-water mixture from the appropriate storage treatment (50% back-slopping). The fermented treatments were sampled after 1 week, and then every second week throughout the trial.

### Grain inoculation

Prior to inoculation, the yeast starter culture was rehydrated with peptone water (Bacteriological peptone 2 g l^−1^; Merck, KGaA., Darmstadt, Germany). The LAB starter culture was pre-grown in 50 ml of yeast extract–peptone–sucrose broth (YPS: yeast extract, 10 g l^−1^ (Oxoid Ltd., Basingstoke, Hampshire, England); bacteriological peptone, 20 g l^−1^ [Oxoid Ltd., Basingstoke, Hampshire, England); and sucrose, 20 g l^−1^ (VWR International Ltd., Poole, England)] at 30°C for 48 h. The yeast and LAB starter culture suspensions, calculated to inoculate 1 × 10^5^ cells/g grain (wet weight) and 2 × 10^5^ cells/g grain, respectively, were sprayed onto the grain post-crimping and mixed thoroughly. The cell numbers were determined by counting in a Bürker chamber. LAB and yeast were enumerated (cfu) and representative isolates were identified to species level in order to verify the inoculation.

### Analytical methods

Grain water activity (*a*_w_) was analyzed using an AquaLab CX-2 (Decagon Devices, Inc., Pullman, Washington, USA) at 22°C. The pH of the liquid obtained after mixing aliquots of 20 g cereal grain for microbial analysis (see Quantification of Microbial Colony Forming Units) was measured using a Laboratory pH meter (Radiometer, Copenhagen, Denmark).

### Quantification of microbial colony forming units

Triplicate aliquots (20 g) of the grain or feed samples were diluted with 180 ml sterile peptone water, supplemented with 0.15 g l^−1^ Tween 80 (Merck, OHG., Schuchardt, Hohenbrunn, Germany), and individually homogenized for 120 s at normal speed in a Stomacher 400 Laboratory blender (Seward Medical, London, UK). Each homogenate was serially diluted in peptone water and spread onto various solid culture media, selective for yeasts, molds, LAB and *Enterobacteriaceae* (malt extract agar, dichloran-glycerol agar, de Man Rogosa Sharp (MRS) agar and violet red bile agar, respectively) as previously described (Lyberg et al., 2008; Olstorpe et al., 2010b). Plates for quantification of molds contained 100 μg ml^−1^ chloramphenicol and 100 μg ml^−1^ cycloheximide, to suppress bacterial and yeast growth, respectively (Druvefors et al., 2002).

### Yeast and lab strain conservation

From each sampling occasion, 10 LAB colonies and 10 yeast colonies were selected randomly from the quantification plates corresponding to the four storage and four fermentation treatments. Isolates were conserved in frozen stocks at −80°C, as previously described (Olstorpe et al., 2008).

### Identification of microorganisms

The LAB and yeast isolates selected above were placed into species groups based on genotypic finger printing using the microsatellite primer (GTG)_5_,from which representative strains were identified by sequencing the 16S rRNA-gene or the D1D2-region of the 26S rRNA-gene, for bacteria and yeasts, respectively, as previously described (Olstorpe et al., 2008, 2010a,b). Briefly, yeast DNA was extracted by boiling a single colony in nuclease-free water, from which 1 μL was used as template in PCR; colony PCR was performed for LAB isolates (Leong et al., 2012). All PCR amplifications were carried out using illustra™ PuReTaq™ Ready-To-Go™ PCR Beads according to the recommendations of the supplier (GE Healthcare, Buckinghamshire, UK). Genotypic fingerprints were analyzed using GelCompar II V4 software (Applied Maths, Kortrijk, Belgium) by creating a dendrogram with settings according to Olstorpe et al. (2008). From the dendrogram, yeasts and LAB at the Pearson correlation of 70% homogeneity were clustered. From each cluster, one representative strain was selected and the appropriate rRNA gene or region thereof was sequenced, thereby determining the species identity of the entire cluster. This was done to evaluate population diversity in the treatments. Purification of PCR-fragments and sequencing was performed at Macrogen, South Korea, and strains were identified by sequence comparison against the databases at EMBL (http://www.ebi.ac.uk) and the Ribosomal database project (http://rdp.cme.msu.edu/) for identification.

Molds were identified as described by Leong et al. (2012). Representative isolates were purified and preliminary identifications performed based on macro- and micro-morphology. DNA was extracted, and selected genes for each isolate were amplified using primers bt2a/bt2b for the β-tubulin gene in *Penicillium* subgenus *Penicillium*, and primers ITS1F/ITS 4 for the rDNA internal transcribed spacer in all other species. Isolates were identified by sequence comparison against various databases, including the Fusarium database (http://isolate.fusariumdb.org/index.php), Genbank and Centraalbureau voor Schimmelcultures (CBS); and accuracy of the sequence-based identifications was confirmed by microscopy.

### Statistical analysis

The procedure Mixed model of SAS (Statistical Analysis System, 2004) was used to analyze pH and quantification data of yeast and LAB. The statistical model for pH included treatment and time as fixed factors. Where the effect of treatment was significant (*P* < 0.05), differences between treatments were tested using least square means (*t*-tests). Microbial counts were log_10_ transformed in order to achieve a better fit to the normal distribution. Interactions were included into the model, although removed from the model when not significant (*P* > 0.05). The statistical model for comparing microbial counts in the moist treatments (D, M, W, and WLAB) included treatment and day of cultivation as fixed factors with two-way interactions. In the statistical design for yeast, containers were used as the main plot and day of cultivation within each container was treated as repeated measurements with the covariance modeled by an unstructured process. In the statistical design for LAB, containers were used as the main plot and day of cultivation within each container was treated as repeated measurements with the covariance modeled by an autoregressive process, as in this case an unstructured process could not be estimated and an autoregressive was considered a good approximation. The statistical model for comparing microbial counts of yeast and LAB in the fermented treatments (FD, FM, FW, and FWLAB) included treatment and day of cultivation as fixed factors with two-way interactions. In the statistical design, containers were used as the main plot and day of cultivation within each container was treated as repeated measurements with the covariance modeled by an unstructured process. Statistically analyzed quantification results are presented as Log_10_ least-square means.

## Results

### Airtight storage of moist barley

Barley was harvested, crimped, and airtight stored in plastic barrels. Four treatments were performed: an uninoculated control (treatment M), storage with inoculated *W. anomalus* (treatment W) and storage with inoculated *W. anomalus* and LAB-consortium (treatment WLAB). Samples for determining grain moisture and pH, and to quantify the microbial populations were taken after inoculation, and 7–19 weeks after storage start.

#### Grain moisture and pH

The average water activity in the moist treatments (M, W, and WLAB) decreased from 0.96 at harvest to 0.85 at week 19. The moist treatments had a pH just over 6.0 at harvest and decreased to an average of 4.6 after 6 weeks. The average pH for each treatment from week 6 to week 19 differed (*p* < 0.05) from 4.73 in M, to 4.55 in W and 4.44 in WLAB.

#### Microbial quantification

The numbers of colony forming units (cfu) for Yeast and LAB (LS means of the log_10_ values of cfu g^−1^ grain) as well as cfu for *Enterobacteriaceae* and molds are shown in Table 1. The inoculation of *W. anomalus* was calculated to yield 10^5^ cfu g^−1^ grain but the determined value was approximately 10^4^ cfu g^−1^ grain in the inoculated treatments. Yeast cfu increased during initial fermentation to about 10^7^ cfu g^−1^ grain in W and WLAB treatment. After the initial fermentation, yeast cfu in treatment W and WLAB decreased after cultivation week 13, and yeast cfu in WLAB was below detection level (<100 cfu g^−1^ grain) in weeks 15 and 19. Colony forming units of LAB were lower in the yeast inoculated treatments than in the control M from week 13 onwards. At harvest, *Enterobacteriaceae* were present on the grain at approximately 10^8^ cfu g^−1^ grain; after 6 weeks of storage no *Enterobacteriaceae* were detected (<10 cfu g^−1^ grain) in the moist treatments, whereas *Enterobacteriaceae* were present on the dry grain (D) at approximately 6.5 Log_10_ cfu g^−1^ grain throughout the trial. At harvest, mold counts were around 10^3^ cfu g^−1^ grain in the moist treatments, but after 7 weeks of storage, no molds were detected (< 100 cfu g^−1^ grain) in treatments W and WLAB. Mold counts in treatment M were below the detection limit after 11 weeks. Molds were present on the dry grain at approximately 10^4^ cfu g^−1^ grain throughout the trial.

**Table 1:** **Microbial quantification of Yeast, Lactic acid bacteria (LAB), *Enterobacteriaceae* and Mold in dried crimped barley (D); moist crimped barley (M); moist crimped barley inoculated with *Wickerhamomyces anomalus* (W); moist crimped barley inoculated with *Wickerhamomyces anomalus* and LAB starter culture (WLAB)**.

**Yeast^1^**	**Week**							
	**0**	**7**	**9**	**11**	**13**	**15**	**17**	**19**
D^2^	3.85	5.87	4.41^a^	3.58^a^	4.86^a^	4.67^a^	5.16^a^	5.51^a^
M	3.85	6.38	7.47^b^	5.04^b^	5.47^a^	5.42^b^	5.32^ab^	b.d.^3^
W	4.0	7.08	7.82^b^	8.01^c^	7.40^b^	6.59^c^	6.09^c^	3.39^b^
WLAB	4.24	6.77	8.53^c^	7.80^c^	7.24^b^	b.d.^3^	5.33^ab^	b.d.^3^
**LAB^4^**								
D^2^	6.77	5.97	3,46	3,82	b.d.^3^	b.d.^3^	b.d.^3^	b.d.^3^
M	6.77	8.07	7.43	7.42	7.43^a^	7.23^a^	6.87^a^	7.44^a^
W	5.54	8.66	7.16	7.62	5.65^b^	6.46^b^	6.46^a^	6.01^b^
WLAB	6.27	7.98	7.33	7.64	5.85^b^	6.13^b^	5.40^b^	5.71^b^
**ENTEROBACTERIACEAE^5^**								
D^2^	8.27	5.63	6.54	6.69	6.52	6.7	6.54	6.54
M	8.27	b.d.^3^	b.d.^3^	b.d.^3^	b.d.^3^	b.d.^3^	b.d.^3^	b.d.^3^
W	7.49	b.d.^3^	b.d.^3^	b.d.^3^	3	b.d.^3^	b.d.^3^	b.d.^3^
WLAB	7.71	b.d.^3^	b.d.^3^	b.d.^3^	b.d.^3^	b.d.^3^	b.d.^3^	b.d.^3^
**MOLD^5^**								
D^2^	3.7	3.26	4.28	3.75	2.43	3.56	3.17	4.03
M	3.7	2.72	5.87	b.d.^3^	b.d.^3^	b.d.^3^	b.d.^3^	b.d.^3^
W	3.41	b.d.^3^	b.d.^3^	b.d.^3^	2.22	b.d.^3^	b.d.^3^	b.d.^3^
WLAB	2.96	b.d.^3^	b.d.^3^	b.d.^3^	b.d.^3^	b.d.^3^	b.d.^3^	b.d.^3^

*Samples were taken after 7 weeks and then consecutively every 2 weeks until week 19*.

1 *Values within columns with different superscript letters (a, b, c) differ significantly (p < 0.05/48 = 0.001), interaction between treatment and week p < 0.0211. Presented as Log_10_ LS means*.

2 *Treatment D was generated by drying grain from the same batch as for treatment M (moist crimped)*.

3 *Below detection level (for yeasts and LAB 100 cfu g^−1^ grain, for molds 10 cfu g^−1^ grain)*.

4 *Values within columns with different superscripts differ significantly (p < 0.05/24 = 0.002), interaction between treatment and week p < 0.001l. Presented as Log_10_ LS means*.

5 *No statistical analysis, presented as mean (n = 3) Log_10_ cfu g^−1^ grain, standard deviation was <1 for all values*.

#### Identified microbial species

Yeast and LAB species composition is shown in Tables 2, 3, respectively. Identification of yeast species at harvest showed that all moist treatments, including M (uninoculated), were completely dominated by *W. anomalus* (*n* = 10). After 7 weeks of storage, *W. anomalus* prevailed in the W treatment and remained dominant until week 15, at which point the dominating species completely shifted to *Pichia kudriavzevii* (formerly known as *Issatchenkia orientalis*). In M, *P. kudriavzevii* and *W. anomalus* were the only species present with *P. kudriavzevii* being isolated somewhat more frequently. In WLAB, the yeast population fluctuated between the same two species. The yeast species composition in D was quite diverse, with a comparatively high number of basidiomycetes (*Cryptococcus* spp. *Rhodotorula graminis*, *Sporobolomyces ruberrimus*), which were not found in the other treatments. Initially dominant LAB species in treatment M and W were *L. plantarum* and *P. pentosaceus*. Over time, no single species was dominant as diversity increased in the control M, while treatment W became mainly dominated by *L. plantarum*. Treatment WLAB, inoculated with LAB starter culture, was dominated by *L. plantarum* at all sampling points. The molds at harvest mainly comprised typical field flora, such as: *Mucor circinelloides*, *Sarocladium strictum*, and *Cladosporium* sp., with *S. strictum* being the dominant species (approximately 50% of all colonies on the quantification plates). After 7 weeks of storage, no molds were found in WLAB treatment. The W treatments were mold free at all sampling points except for week 13 when *Penicillium roqueforti* was isolated from the barrel open at the time. Molds were, however, detected in treatment M, which contained *Paecilomyces lilacinus* (60%), *Rhizomucor variabilis* var. *regularior* (20%) and *Cladosporium* sp. (20%). In week 9 the mold population in M had shifted entirely to *P. roqueforti*. Treatment D was initially dominated by *Fusarium tricinctum* (30%), *Lecanicillium* sp. (40%), *Cladosporium* sp. (15%) and *Penicillum griseofulvum* (15%). Over time *Lecanicillium* sp. became dominant over *Cladosporium* sp. and *P. roqueforti*.

**Table 2:** **Yeast species isolated from dried crimped barley (D); moist crimped barley (M); moist crimped barley inoculated with *Wickerhamomyces anomalus* (W); moist crimped barley inoculated with *Wickerhamomyces anomalus* and LAB starter culture (WLAB)**.

**Treatment**	**D**							**M**							**W**							**WLAB**						
**Week**	**7**	**9**	**11**	**13**	**15**	**17**	**19**	**7**	**9**	**11**	**13**	**15**	**17**	**19**	**7**	**9**	**11**	**13**	**15**	**17**	**19**	**7**	**9**	**11**	**13**	**15**	**17**	**19**
*Acremonium strictum*							2							b.d.^1^												b.d.^1^		b.d.^1^
*Aureobasidium pullulans*				3		1								b.d.^1^												b.d.^1^		b.d.^1^
*Debaryomyces hansenii*		3	1	1	5	3								b.d.^1^												b.d.^1^		b.d.^1^
*Pichia fermentans*						3								b.d.^1^												b.d.^1^		b.d.^1^
*Pichia kudriavzevii*					2			5	4	5	8	8	7	b.d.^1^					10	10	8	7		2		b.d.^1^	10	b.d.^1^
*Saccharomyces castellii*														b.d.^1^									2			b.d.^1^		b.d.^1^
*Saccharomyces cerevisiae*	3													b.d.^1^												b.d.^1^		b.d.^1^
*Wickerhamomyces anomalus*	3		2					5	6	5	2	2	3	b.d.^1^	10	10	10	10			2	3	8	8	10	b.d.^1^		b.d.^1^
*Cryptococcus magnus*							1							b.d.^1^												b.d.^1^		b.d.^1^
*Cryptococcus victoria*				1		1	4							b.d.^1^												b.d.^1^		b.d.^1^
*Rhodotorula graminis*			7	2	3	2	1							b.d.^1^												b.d.^1^		b.d.^1^
*Sporobolomyces ruberrimus*	4	7		3			2							b.d.^1^												b.d.^1^		b.d.^1^

*Samples were taken after 7 weeks of storage and then consecutively every 2 weeks until week 19. Numbers in columns reflect the species frequency among 10 identified isolates per sampling occasion. Species were identified by D_1_D_2_ rDNA gene sequencing using primers NL1/NL4*.

1 *Below detection level (100 cfu g^−1^ grain)*.

**Table 3:** **Lactic acid bacteria (LAB) species isolated from dried crimped barley (D); moist crimped barley (M); moist crimped barley inoculated with *Wickerhamomyces anomalus* (W); moist crimped barley inoculated with *Wickerhamomyces anomalus* and LAB starter culture (WLAB)**.

**Treatment**	**D**							**M**							**W**							**WLAB**						
**Week**	**7**	**9**	**11**	**13**	**15**	**17**	**19**	**7**	**9**	**11**	**13**	**15**	**17**	**19**	**7**	**9**	**11**	**13**	**15**	**17**	**19**	**7**	**9**	**11**	**13**	**15**	**17**	**19**
*Lactobacillus curvatus*				b.d.^1^	b.d.^1^	b.d.^1^	b.d.^1^	2																				
*Lactobacillus brevis*				b.d.	b.d.	b.d.	b.d.					2	1	4			1	1		6							1	
*Lactobacillus buchneri*				b.d.	b.d.	b.d.	b.d.						2	1														
*Lactobacillus coryniformis*				b.d.	b.d.^1^	b.d.^1^	b.d.^1^						1	1					4	1						2		2
*Lactobacillus crustorum*				b.d.^1^	b.d.^1^	b.d.^1^	b.d.^1^																	2				
*Lactobacillus paralimentarius*				b.d.^1^	b.d.^1^	b.d.^1^	b.d.^1^																	1		1		
*Lactobacillus plantarum*	5	6	4	b.d.^1^	b.d.^1^	b.d.^1^	b.d.^1^	4	1	10	4	6	3	1	6	9	9	9	6	2	10	10	10	7	10	7	9	8
*Leuconostoc citreum*	2	1	4	b.d.^1^	b.d.^1^	b.d.^1^	b.d.^1^																					
*Leuconostoc mesenteroides*			2	b.d.^1^	b.d.^1^	b.d.^1^	b.d.^1^																					
*Pediococcus parvulus*				b.d.^1^	b.d.^1^	b.d.^1^	b.d.^1^							1														
*Pediococcus cellicola*				b.d.^1^	b.d.^1^	b.d.^1^	b.d.^1^						1							1								
*Pediococcus pentosaceus*	3	3		b.d.^1^	b.d.^1^	b.d.^1^	b.d.^1^	4	9		6	2	2	2	4	1												
*Weissella paramesenteroides*				b.d.^1^	b.d.^1^	b.d.^1^	b.d.^1^																					

*Samples were taken after 7 weeks of storage and then consecutively every 2 weeks until week 19. Numbers in columns reflect the species frequency among 10 identified isolates per sampling occasion. Species were identified by rDNA gene sequencing using primers 16Ss/16Sr*.

1 *Below detection level (100 cfu g^−1^ grain)*.

### Feed fermentations

After 6 weeks of storage, feed fermentations were prepared from the treated grain (D, M, W, WLAB) by mixing it with water to a dry matter content of 30% (generating treatments FD, FM, FW, and FWLAB). To FWLAB, additional LAB-starter culture was added at the beginning of fermentation, as recommended by the provider. We assumed that addition of *W. anomalus* was not required, as it typically was dominant during airtight storage in all our previous experiments (Olstorpe and Passoth, 2011). The mixtures were initially incubated for 1 week; thereafter, 50% was removed daily for feeding, and replaced by new material from the storage systems. The average pH in the fermented treatments FM, FW and FWLAB from week 7–19 was 4.08 (±0.04). The fermented dry treatment (FD) had a lower pH (*p* < 0.05) of 3.84.

#### Microbial quantification

Cfu for Yeast and LAB (Log_10_ LS means cfu g^−1^ grain) as well as cfu for *Enterobacteriaceae* and molds (Log_10_ mean cfu g^−1^ grain) are shown in Table 4. Cfu of yeasts were above Log_10_ 7 in all treatments at all cultivations, and cfu of LAB fluctuated between 8 and 9.7 log_10_ units.

**Table 4:** **Microbial quantification (Log_10_ cfu g^−1^ grain) of Yeast, Lactic acid bacteria (LAB), *Enterobacteriaceae* and Mold in Fermented dried crimped barley (FD); fermented moist crimped barley (FM); fermented moist crimped barley storage-inoculated with *Wickerhamomyces anomalus* (FW); fermented moist crimped barley storage-inoculated with *Wickerhamomyces anomalus* and LAB starter culture; additional LAB starter culture inoculated at the beginning of fermentation (FWLAB)**.

**Yeast^1^**	**Week**						
	**0^2^**	**2**	**4**	**6**	**8**	**10**	**12**
FD	7.35^a^	7.39	7.48	7.19	7.64	7.34	7.48^a^
FM	7.36^a^	7.54	7.39	7.36	7.14	7.40	7.78^ab^
FW	7.55^a^	7.57	7.32	7.00	7.55	7.46	7.89^b^
FWLAB	8.71^b^	7.61	7.41	7.52	7.60	7.49	7.50^a^
**LAB^3^**							
FD	8.81^a^	9.34	9.44^a^	8.76^ab^	9.47^a^	9.31	9.64^a^
FM	9.36^ab^	8.99	8.66^b^	9.15^a^	8.84^b^	9.07	9.02^ab^
FW	9.17^ab^	8.81	8.56^b^	8.49^b^	8.94^b^	9.30	9.12^ab^
FWLAB^4^	9.70^b^	8.30	8.15^b^	8.81^ab^	8.94^b^	8.97	8.86^b^
**ENTEROBACTERIACEAE^5^**							
FD	2.45	b.d.^6^	b.d.^6^	b.d.^6^	b.d.^6^	b.d.^6^	b.d.^6^
FM	5.75	b.d.^6^	b.d.^6^	b.d.^6^	b.d.^6^	b.d.^6^	b.d.^6^
FW	b.d.^6^	b.d.^6^	b.d.^6^	b.d.^6^	b.d.^6^	b.d.^6^	b.d.^6^
FWLAB	b.d.^6^	b.d.^6^	b.d.^6^	b.d.^6^	b.d.^6^	b.d.^6^	b.d.^6^
**MOLD^5^**							
FD	4.75	b.d.^6^	2.86	2.2	2.30	2.43	2.48
FM	2.65	4.62	5.42	5.43	5.23	b.d.^6^	b.d.^6^
FW	b.d.^6^	b.d.^6^	b.d.^6^	4.00	5.16	b.d.^6^	b.d.^6^
FWLAB	b.d.^6^	b.d.^6^	b.d.^6^	4.73	2.67	2.6	b.d.^6^

*Samples were taken after 1 week of undisturbed fermentation and then consecutively every 2 weeks until week 12 of fermentation (equivalent to week 19 of storage trial)*.

1 *Values within a column with different superscript letters (a, b) differ significantly (p < 0.05/42 = 0.0012), Interaction between treatment and week: p < 0.0204. Presented as Log_10_ LS means*.

2 *Start of feed-outtake and backslopping; backslopping from airtight stored grain, at this time stored for 7 weeks*.

3 *Values within a column with different superscripts differ significantly (p < 0.05/42 = 0.0012), Interaction between treatment and week: p < 0.08. Presented as Log_10_ LS means*.

4 *Additional LAB starter culture was grown in yeast extract–peptone–sucrose (YPS) broth at 30°C for 48 h, and added to the FWLAB treatment to ensure viability of the culture*.

5 *No statistical analysis, presented as mean (n = 3) Log_10_ cfu g^−1^ grain, standard deviation was < 1 for all values*.

6 *Below detection level (for yeasts and LAB 100 cfu g^−1^ grain, for molds 10 cfu g^−1^ grain)*.

No *Enterobacteriaceae* were detected in treatments FW and FWLAB after 1 week of fermentation; in FD and FM they prevailed longer and were detected until 3 weeks of fermentation. Molds were present in FD at all sampling points. In FM, mold counts were below detection after 10 weeks. No molds were detected in FW and FWLAB during the first 3 samplings, but after 6 weeks, some mold growth was observed in both treatments. In treatment FW, mold counts were below detection limit again in week 10, and in FWLAB, in week 12.

#### Identified microbial species

Yeast and LAB species composition during fermentation are shown in Tables 5, 6, respectively. Three major yeast species were found in all four treatments, *K. exigua*, *W. anomalus*, and *P. kudriavzevii*. Interestingly, *K. exigua* was not identified among the isolates from the grain storage (Table 5). This species was not identified during the first samplings, but became dominant during the course of fermentation in all treatments. *W. anomalus* was present from the beginning in all treatments, but its proportion decreased over the duration of the experiment. *P. kudriavzevii* was only occasionally found in FD, but was present in all treatments derived from moist stored grain. Similar to *W. anomalus, P. kudriavzevii* was present at relatively high proportions in the beginning of the fermentations, but decreased toward the end. Among the LAB, *L. plantarum* was the most frequently isolated species in all treatments, which correlates with its presence in the stored grain (Table 6). However, several minor species in grain storage were quite abundant in feed fermentation. In FD, *Lactobacillus paralimentarius* was found at high proportions especially at the end of the fermentation. With moist stored grain as the starting material, the LAB-population became more diverse toward the end of the fermentation: *L. paralimentarius*, *Lactobacillus nantensis*, or *Lactobacillus crustorum* joined *L. plantarum* in representing a higher proportion of the identified species. *P. pentosaceus* was less prominent than one might expect, given that it comprised a considerable proportion of the LAB-flora in the stored grain and was even re-inoculated in the FWLAB treatment. No *P. acidilactici* was detected in any of the fermented samples. The mold population in the feed fermentations varied to some extent. When mold counts were elevated in FW and FWLAB, only *P. roqueforti* was found. In FD and FM, the initial species isolated were *Geotrichum candidum* (100%) after 1 week fermentation in FD, and *Rhizomucor variabilis* var. *regularior* (100%) in FM. For the next 6 weeks, *P. roqueforti* was the major species (95–100%) of all isolates. Minor species (5% of the total isolates) included *Epicoccum nigrum* (FM, 3 weeks) and *Cladosporium* sp. (FD, 5 weeks). In FD after 7 weeks, there was a consortium of *Penicillium palitans* (40%) and *R. variabilis* var. *regularior* (60%), and after 9 weeks (end of fermentation) *P. palitans* comprised 100% of the mold population.

**Table 5:** **Yeast species isolated from fermented dried crimped barley (FD); fermented moist crimped barley (FM); fermented moist crimped barley storage-inoculated with *Wickerhamomyces anomalus* (FW); fermented moist crimped barley storage-inoculated with *Wickerhamomyces anomalus* and LAB starter culture; additional LAB starter culture inoculated at the beginning of fermentation (FWLAB)**.

**Treatment**	**FD**							**FM**							**FW**							**FWLAB**						
**Week**	**0**	**2**	**4**	**6**	**8**	**10**	**12**	**0**	**2**	**4**	**6**	**8**	**10**	**12**	**0**	**2**	**4**	**6**	**8**	**10**	**12**	**0**	**2**	**4**	**6**	**8**	**10**	**12**
*Debaryomyces hansenii*		1																										
*Kazachstania exigua*		4	9	6	10	8	10		1	8	10	9	10	10				3	7	10	10				7	8	10	10
*Pichia fermentans*						2																						
*Pichia kudriavzevii*			1					9	3	1		1			9	6	2	3				10	2	1				
*Pichia membranifaciens*	6							1																				
*Saccharomyces castellii*		4							1																			
*Wickerhamomyces anomalus*	4	1		4					5	1					1	4	8	4	3				8	9	3	2		

*Samples were taken after 1 week of undisturbed fermentation and then consecutively every 2 weeks until week 12 (equivalent to week 19 of storage trial). Numbers in columns reflect the species frequency among 10 identified isolates per sampling occasion. Species were identified by D_1_D_2_ rDNA gene sequencing using primers NL1/NL4*.

**Table 6:** **Species of LAB isolated from fermented dried crimped barley (FD); fermented moist crimped barley (FM); fermented moist crimped barley inoculated with *Wickerhamomyces anomalus* (FW); fermented moist crimped barley inoculated with *Wickerhamomyces anomalus* and LAB starter culture; additional LAB starter culture inoculated at the beginning of fermentation (FWLAB)**.

**Treatment**	**FD**							**FM**							**FW**							**FWLAB**						
**Week**	**0**	**2**	**4**	**6**	**8**	**10**	**12**	**0**	**2**	**4**	**6**	**8**	**10**	**12**	**0**	**2**	**4**	**6**	**8**	**10**	**12**	**0**	**2**	**4**	**6**	**8**	**10**	**12**
*Lactobacillus brevis*				1							1	3	2		2			1	2	1	1				1	1		2
*Lactobacillus crustorum*			6	1						3		1	2			2		3	3	4	1			1	1	4	5	
*Lactobacillus curvatus*	5																											
*Lactobacillus kimchii*					1															1							1	
*Lactobacillus nantensis*											3			6							6							1
*Lactobacillus parabrevis*																												2
*Lactobacillus paralimentarius*			2	6	8	10	10			1	1	1	1	4							2							1
*Lactobacillus plantarum*	3	10	2	2	1			10	10	5	4	2	3		7	8	10	5	3	3		9	9	7	8	4	4	2
*Pediococcus cellicola*																		1										
*Pediococcus ethanolidurans*											1	1							1									
*Pediococcus parvulus*										1		2							1					2				1
*Pediococcus pentosaceus*	2												2		1					1		1	1			1		

*Samples were taken after 1 week of undisturbed fermentation and then consecutively every 2 weeks until week 12 (equivalent to week 19 of storage trial). Numbers in columns reflect the species frequency among 10 identified isolates per sampling occasion. Species were identified by rDNA gene sequencing using primers 16Ss/16Sr*.

## Discussion

This study intended to demonstrate the utility of a starter culture for airtight storage of moist grain that can even impact a subsequent feed fermentation performed with this grain. If the same starter culture can be used for both moist airtight storage and feed fermentation, handling of the microbes is simplified and thus it is more attractive for the farmer.

Using microbes that have been isolated from both moist grain preservation and feed fermentation for airtight storage of moist grain indeed improved the hygiene of the feed. Microbes that can jeopardize feed quality—molds and *Enterobacteriaceae*—were reduced by moist storage. Adding starter cultures had at least an effect on how rapidly molds were inhibited, because in the non-inoculated treatment, molds were still detected after 9 weeks of storage. Interestingly, molds and *Enterobacteriaceae* obviously survived drying the material to moisture content of 13%, as at all samplings of the dried crimped grain, considerable amounts of these microbes were observed. Especially for the latter, this is a surprising result, as *Enterobacteriaceae* do not tolerate low water activities and develop metabolic lesions in dry environments. However, it has been observed that if the injured cells are introduced into an environment with a conducive water activity, they will recover and proliferate once again (Mossel and Ratto, 1970). Our results indicate that moist airtight storage of grain may represent a storage technology that is even safer than the commonly used procedure of drying the grain, if all parameters are carefully controlled. Parameters that efficiently inhibit *Enterobacteriaceae* are low pH (Adams and Hall, 1988), the airtight environment with high levels of CO_2_ (Mazzoni et al., 2001) and the presence of a yeast such as *W. anomalus*, which can inhibit this group of bacteria (Olstorpe et al., 2010b, 2012).

Feed fermentation further reduced *Enterobacteriaceae*, which were only detected at the first sampling point, and furthermore, only from the non-inoculated storage experiments, i.e., D and M. Molds levels were also low, being close to or below the detection limit at most of the sampling points. Adding starter cultures to the stored grain resulted in a stronger inhibition of molds compared to fermentation of non-inoculated material, although this inhibition was not complete and molds were detected in FW at the 6 and 8 week sampling points, and in FWLAB at 6, 8, and 10 weeks of fermentation. The reasons for this intermediate increase in mold cfu are not clear. When molds were initially detected (week 6), a shift in the dominance of the yeast species from *W. anomalus*/*P. kudriavzevii* to *K. exigua* was also observed. At the same time, the LAB population changed from a dominance of *L. plantarum* to a more diverse population. One may speculate that these changes reflected an imbalance in the microbial population, which then opened a niche for molds to grow in the system. Nevertheless, the number of molds was at most sampling points rather low, and thus it can be concluded that airtight moist grain storage in connection with feed fermentation generally improves feed hygiene.

Identification of yeasts and LAB provided some unexpected results. During airtight storage, *W. anomalus* was finally overgrown by *P. kudriavzevii*. This yeast must have been present in the storage system from beginning, because it was also present in the non-inoculated treatment. Outcompetition of inoculated strains by those present in the system has been observed several times in more or less open fermentation systems, such as wine making (Vaughan-Martini and Martini, 1995). However, the *W. anomalus* strain used in this study had been originally isolated from cereal grain and was several times demonstrated to be competitive in airtight storage (Olstorpe et al., 2010b; Olstorpe and Passoth, 2011). This indicates that either the *P. kudriavzevii* strain at this farm was especially competitive, or that the competitive and/or survival capacities of the *W. anomalus* strain were compromised by the large scale, industrial nature of inoculum production. Nevertheless, the succession by *P. kudriavzevii* obviously did not impact the storage stability, as molds and *Enterobacteriaceae* were still below detection limit (with one exception in W after 13 weeks, where molds were slightly above detection level). The other unexpected result was that *P. kudriavzevii*, having become dominant in stored grain, was itself overgrown in the feed fermentation by another non-inoculated species, *Kazachstania exigua*. As this yeast was never found among the isolates from grain storage, it is likely that it was present on the equipment for feed fermentation.

In the feed fermentations, cfu counts of yeast remained on a similar level in all treatments at all sampling points, however, the yeast species composition changed over time. Similar observations have also been obtained in previous fermentation trials, and hence cfu counts alone are poor indicators of population stability (Olstorpe et al., 2008, 2010a).

Of the three LAB species in the starter culture, *P. acidilactici*, *P. pentosaceus*, and *L. plantarum*, only *L. plantarum* reached dominance both in the storage system and in the fermented feed. This species seems to be naturally present on the cereal grain, as it also was found in non-inoculated material. *P. pentosaceus* was also present on both the inoculated and non-inoculated material, but only at low levels. In the fermented feeds, *L. plantarum* became less dominant at the later sampling points (from week 13 to 15) and the diversity of LAB generally increased. This happened in spite of its daily re-introduction into the fermentations, via daily replacement of fed-material with corresponding moist stored grain, i.e., grain on which *L. plantarum* was dominant. It is possible that this phenomenon is also correlated to the observed shift in yeast species dominance. However, this somewhat contrasts the findings of Gobbetti et al. (1994) who reported an increase in cell yield and lactic acid production by *L. plantarum* when it was used together with *K. exigua* in sourdough. Damiani et al. (1996) proposed a mix of *K. exigua* and *L. plantarum* as a starter for sour dough. Our results indicate that still many aspects of both airtight storage of moist grain and pig feed fermentation are not well understood.

Nevertheless, moist grain storage coupled with fermentation showed several positive effects. Airtight stored moist grain had fewer *Enterobacteriaceae* than dry grain, and thus moist airtight storage seems to improve feed hygiene. In general, fewer molds and *Enterobacteriaceae* were found in fermented material. The material was also used for feeding animals in an ongoing feed trial, and no problems with the feed were observed (manuscript in preparation). Adding starter cultures had some effect on the microbial populations during both storage and feed fermentation, and it was obviously possible to influence the microbial population in the fermented feed by adding appropriate starter organisms to the grain storage system. This may open up a way to simplify feed handling processes, which in turn will increase the acceptance of such measures for feed improvement. However, many factors determining dominance in storage- and feed fermentation systems are still unknown, and the microbial population present on the equipment may strongly influence the final output of feed storage and fermentation. The microbial diversity on farm equipment may represent an untapped niche to isolate and screen for advantageous and competitive strains. Thereafter, cultivation and formulation methods can be developed to produce those strains as efficient starter cultures for integrated storage and feed fermentation processes.

### Conflict of interest statement

The authors declare that the research was conducted in the absence of any commercial or financial relationships that could be construed as a potential conflict of interest.
